# Influence of Incubation Time on Ortho-Toluidine Blue Mediated Antimicrobial Photodynamic Therapy Directed against Selected *Candida* Strains—An In Vitro Study

**DOI:** 10.3390/ijms222010971

**Published:** 2021-10-11

**Authors:** Rafał Wiench, Joanna Nowicka, Magdalena Pajączkowska, Piotr Kuropka, Dariusz Skaba, Anna Kruczek-Kazibudzka, Anna Kuśka-Kiełbratowska, Kinga Grzech-Leśniak

**Affiliations:** 1 Department of Periodontal Diseases and Oral Mucosa Diseases, Faculty of Medical Sciences in Zabrze, Medical University of Silesia, 40-055 Katowice, Poland; rwiench@sum.edu.pl (R.W.); dskaba@sum.edu.pl (D.S.); anna.kuska@hotmail.com (A.K.-K.); 2 Department of Microbiology, Faculty of Medicine, Wroclaw Medical University, 50-368 Wroclaw, Poland; joanna.nowicka@umed.wroc.pl (J.N.); magdalena.pajaczkowska@umed.wroc.pl (M.P.); 3 Department of Histology and Embryology, Wroclaw University of Environmental and Life Sciences, 50-375 Wroclaw, Poland; piotr.kuropka@upwr.edu.pl; 4 Private Practice, 44-100 Gliwice, Poland; kruczeka00@gmail.com; 5 Laser Laboratory Dental Surgery Department, Medical University of Wroclaw, 50-425 Wroclaw, Poland; 6 Department of Periodontics, School of Dentistry, Virginia Commonwealth University, Richmond, VA 23284, USA

**Keywords:** aPDT, diode laser, TBO, yeasts, oral candidiasis, oral microbiome

## Abstract

(1) Background and the aim: The appropriate incubation time in the antimicrobial photodynamic therapy protocol seems to have a huge impact on the efficacy of this process. This is particularly important in relation to *Candida* strains, due to the size of these cells and the presence of the cell wall. The aims of this study were to determine the optimal incubation time needed for the absorption of toluidine blue by cells of *C. albicans*, *C. glabrata*, *C. krusei* and *C. parapsilosis* using direct observation by optical microscopy, and to evaluate the efficacy of TBO-mediated aPDT on planktonic cells of these strains. (2) Methods: The microscopic evaluation consisted of taking a series of images at a magnification of 600× and counting the % of stained cells. The in vitro effect of TBO-mediated aPDT combined with a diode laser (635 nm, 400mW, 12 J/cm^2^, CW) on the viability of yeast cells with different incubation times was evaluated. (3) Results: The presence of TBO within the cytoplasm was observed in all tested *Candida* strains and at all microscopic evaluation times. However, the highest percentages of cells were stained at 7 and 10 min. The highest % reduction of CFU/mL after TBO-mediated aPDT against *Candida* was obtained for the strain *C. albicans* ATCC 10,231 and it was 78.55%. (4) Conclusions: TBO-mediated aPDT against *Candida* was effective in reducing the number of CFU/mL at all assessed incubation times. However, the most efficient period for almost all strains was 7–10 min.

## 1. Introduction

Photodynamic therapy (PDT) is a kind of light therapy [1] which causes irreversible damage to target cells (diseased host cells or microorganisms) and has been shown to be a potential approach for cancer treatment [2,3,4] and for treating microbial infection induced by Gram-positive and Gram-negative bacteria, including antibiotic-resistant strains [5,6,7,8] as well as yeasts [9,10]. If the cells being destroyed are microorganisms, this form of therapy is called antimicrobial photodynamic therapy (aPDT).

The basis of PDT is the cooperation of three elements: photosensitizer (PS), light, and oxygen. These components are harmless by themselves but combined they lead to selective destruction of pathogenic cells [11]. It is important to take into consideration that different PSs have varying absorption maximums. The PS is usually an organic, aromatic dye molecule capable of delocalizing π electrons and is non-toxic in the absence of light. It is an external chromophore that absorbs light in the red or near-infrared range (600–800 nm); wavelengths >800 nm do not have enough internal photonic energy to induce a photodynamic reaction, while wavelengths <600 nm have less tissue penetration [12]. The antimicrobial effect of a PS is strictly dependent on its physical properties (absorption peak (k_max_), intensity of absorption (e_max_), and quantum yield for singlet oxygen) and chemical parameters (lipophilicity/hydrophilicity balance (logP) and the presence of charged groups that determine the mechanism of cellular uptake). As a rule, hydrophilicity and the presence of charged groups are better for cellular uptake [13].

The light sources used for PDT are typically lasers, light-emitting diodes (LEDs), or lamps with a broad spectrum of wavelengths. Lasers and LEDs seem to be the most appropriate sources of light. They can provide the most exact match to the absorption maximum of a PS, which results in less heating of the tissues and an optimal course of the photodynamic reaction [14]. The absorption peak of the PS changes with the concentration of the aqueous solution, and therefore when selecting a light source for therapy or experiments, particular attention should be paid to this parameter [15].

The interest in the possibility of using aPDT for treatment of oral candidiasis results from the infectious nature of these etiopathogenic factors and the more and more frequent multiple resistance to popular antimycotics [16]. aPDT against *Candida* (*C.*) spp. is more difficult than against bacteria, related to the larger size of fungal cells (on average 25–50 times larger than bacterial cells) and their more complex structure [17]. Particularly important is the additional thick cell wall consisting of glucans, mannans, chitin, and lipoproteins [18,19,20] and the presence of a cell nucleus separated from the cytoplasm by a nuclear membrane. Yeast could also produce antioxidant enzymes such as superoxide dismutase (SOD) and catalase. SOD converts damaging superoxide radicals (O_2_^−^), one type of reactive oxygen species (ROS), to the less damaging hydrogen peroxide (H_2_O_2_) which can be converted into water by catalase [21,22,23]. Moreover, many species of fungi, including *Candida* spp., have specific defense mechanisms against aPDT including inhibition of PS uptake, stimulation of its excretion from the cell, or intracellular metabolism by inducing enzymatic remodeling of PS particles [23]. Therefore, aPDT against yeasts requires application of only selected PS which should also be used in higher concentrations, for longer incubation times, and with higher parameters of the laser physical settings (power density, fluence) [24,25]. One of the most used PS is toluidine blue ortho (TBO) also known as tolonium chloride, a basic metachromatic thiazide dye. Its small size, good solubility in water, cationic form, hydrophilic character, and tendency to form dimers facilitate its binding to microorganisms’ cell membranes [26]. A large difference in affinity for the surface of yeast and host cells provides selectivity, and no DNA damage to keratinocytes in vitro was observed [27,28]. Its potential to kill yeast cells is to damage the cell wall where PS molecules, thanks to their cationic form, create ionic bonds between amino groups on the surface [29,30]. As a result of photoactivation, the oxidation processes change the architecture of the cell so that the TBO reaches the cell membrane and the cytoplasm [24]. These processes require longer contact times of TBO with the surface of the yeast cell wall and membrane so that the large and biochemically complex PS molecules can pass through the narrow *Candida* membrane channels. Practically, this translates into use of a sufficiently long incubation time (pre-irradiation time, PIT) to accumulate a sufficient amount of PS on the surface of the yeast cell and inside the cytoplasm so that the efficacy of TBO-mediated aPDT is as high as possible [14]. The selection of the most efficacious photosensitizer, as well as the laser dosimetry, to be used in the elimination of *Candida* have been the subject of many studies [29,30].

The least studied element of the aPDT procedure for treating candidiasis is the optimal incubation time with the PS. Therefore, the aims of this study were to determine the optimal incubation time needed for the absorption of TBO by cells of selected *Candida* strains using direct observation by optical microscopy, and to evaluate the efficacy of TBO-mediated aPDT on planktonic cells of these strains.

## 2. Results

**Figure 1:**
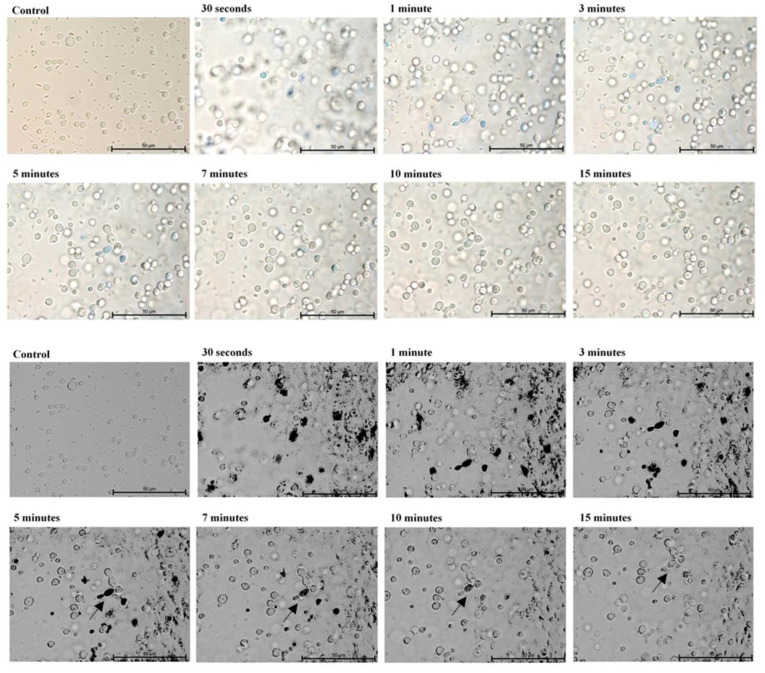
A representative series of original images (two upper rows) and images after isolating indigo and blue colors and converting to black in ImageJ (two lower rows) showing the number of *Candida* *albicans* ATCC 90,028 cells with absorbed ortho-toluidine blue (TBO) at individual times relative to the control. Magnification 600×. Scale bars = 50 µm. Black arrows indicate yeast cells with gradually disappearing dye in the cytoplasm. American Type Culture Collection (ATCC).

**Figure 2:**
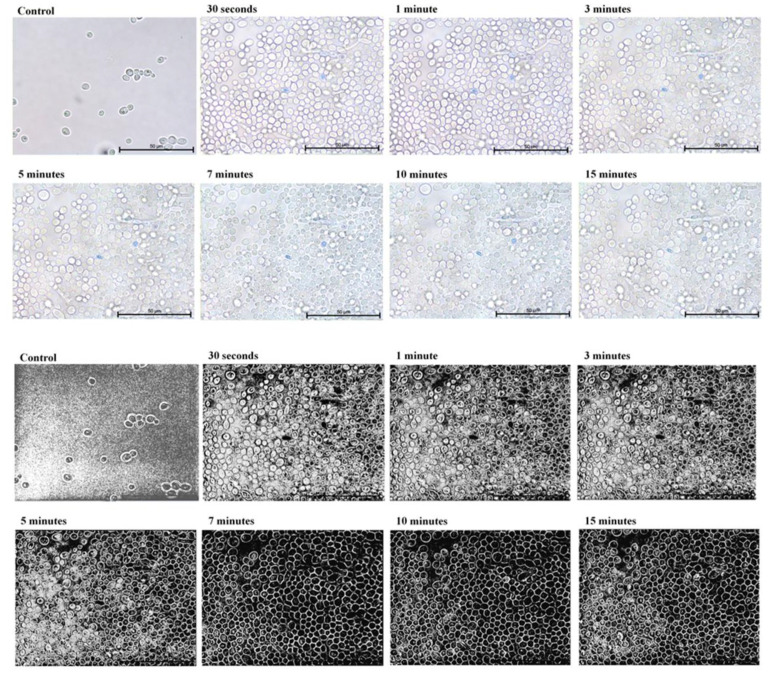
A representative series of original images (two upper rows) and images after isolating indigo and blue colors and converting to black in ImageJ (two lower rows) showing the number of *Candida* *albicans* ATCC 10,231 cells with absorbed ortho-toluidine blue (TBO) at individual times relative to the control. Magnification 600×. Scale bars = 50 µm. American Type Culture Collection (ATCC).

**Figure 3:**
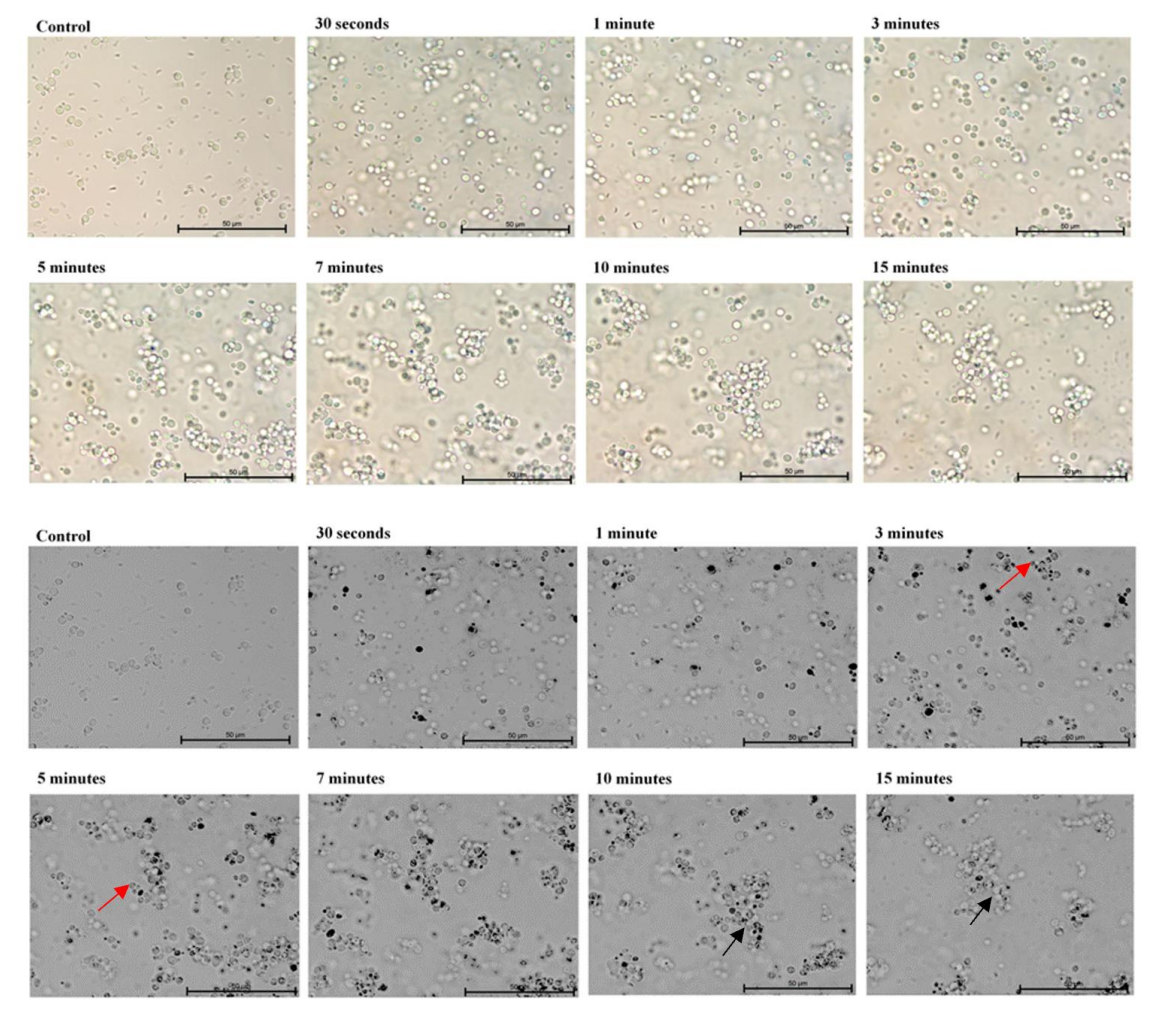
A representative series of original images (two upper rows) and images after isolating indigo and blue colors and converting to black in ImageJ (two lower rows) showing the number of *Candida* *glabrata* ATCC 90,030 cells with absorbed ortho-toluidine blue (TBO) at individual times relative to the control. Magnification 600×. Scale bars = 50 µm. Black arrows indicate yeast cells with gradually disappearing dye in the cytoplasm. Red arrows indicate representative early aggregation structures of *Candida glabrata* ATCC 90,030. American Type Culture Collection (ATCC).

**Figure 4:**
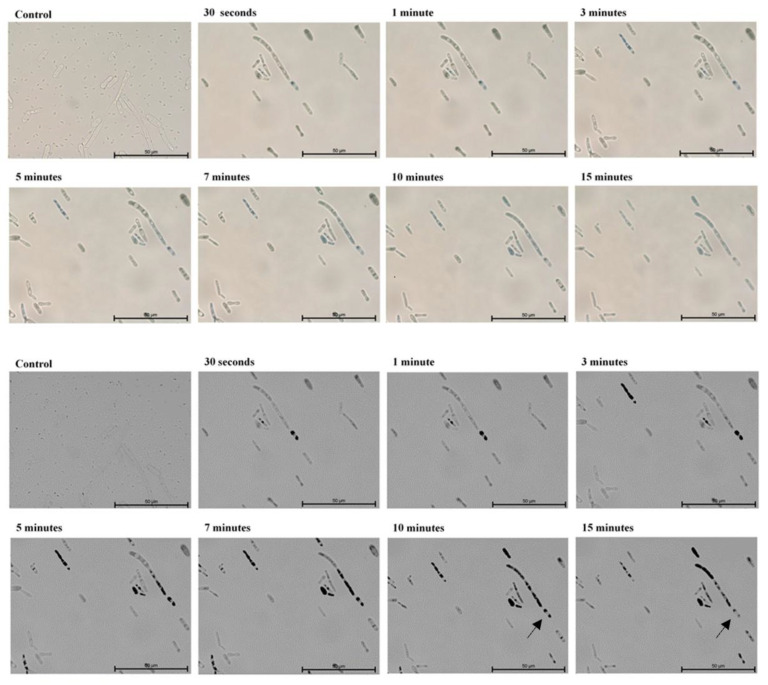
A representative series of original images (two upper rows) and images after isolating indigo and blue colors and converting to black in ImageJ (two lower rows) showing the number of *Candida* *krusei* ATCC 6258 cells with absorbed ortho-toluidine blue (TBO) at individual times relative to the control. Magnification 600×. Scale bars = 50 µm. Black arrows indicate yeast cells with gradually disappearing dye in the cytoplasm. American Type Culture Collection (ATCC).

**Figure 5:**
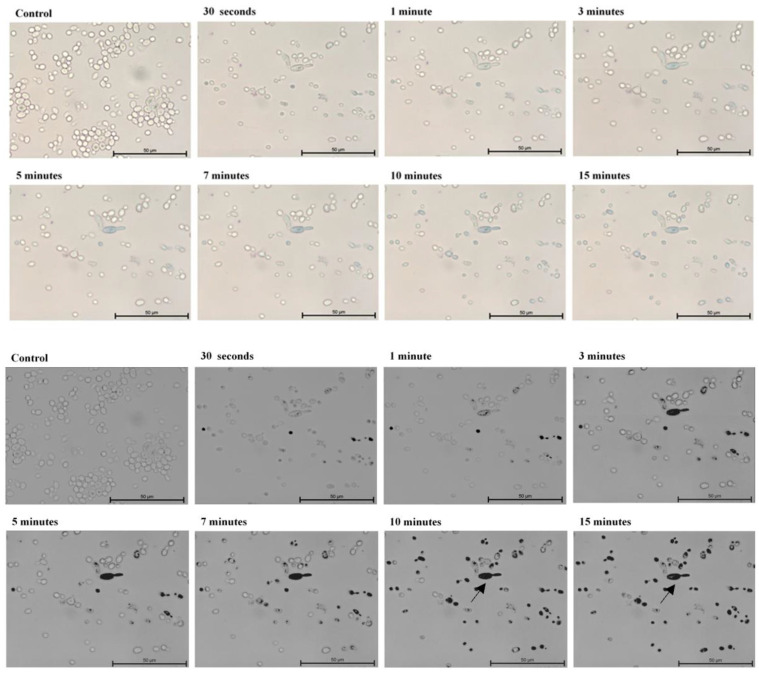
A representative series of original images (two upper rows) and images after isolating indigo and blue colors and converting to black in ImageJ (two lower rows) showing the number of *Candida parapsilosis* ATCC 90,018 cells with absorbed ortho-toluidine blue (TBO) at individual times relative to the control. Magnification 600×. Scale bars = 50 µm. Black arrows indicate yeast cells with gradually disappearing dye in the cytoplasm. American Type Culture Collection (ATCC).

In the first part of the experiment, direct microscopic evaluation of the absorption of TBO by cells of *Candida*, the presence of cells containing photosensitizer was observed in all the strains and at all sampling times. Figure 1, Figure 2, Figure 3, Figure 4 and Figure 5 show an exemplary series of original micrographs for each strain and the same representative series after processing the original images in ImageJ to increase contrast.

**Figure 6:**
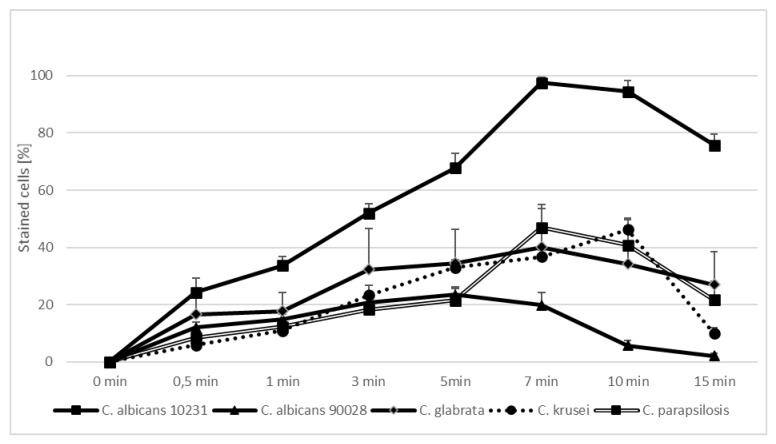
TBO uptake by *Candida albicans* ATCC 10,231, *Candida albicans* ATCC 90,028, *Candida glabrata* ATCC 90,030, *Candida krusei* ATCC 6258 and *Candida parapsilosis* ATCC 90,018 over time (0–15 minutes) by direct observation in an optical microscope. Data are mean values and standard deviations from six replicate experiments. American Type Culture Collection (ATCC).

Descriptive statistics expressed as the percentage of stained cells visible in the field of view of the microscope at particular observation times are presented in Figure 6. Large differences in PS absorption by different strains of the yeast were found. The highest percentage of TBO-containing cells (mean) was observed in *C. albicans* ATCC 10,231 and was −97.53%. *C. parapsilosis* ATCC 90,018 −47.0%, *C. krusei* ATCC 6258 −46.29% and *C. glabrata* ATCC 90,030 −40.15% were recorded consecutively. The lowest result was achieved by *C. albicans* ATCC 90,028 −23.48%. The time required to obtain the maximum adsorption also differed for individual strains and ranged from 5 min for *C. albicans* ATCC 90,028, 7 min for *C. albicans* ATCC 10,231, *C. glabrata* ATCC 90,030, *C. parapsilosis* ATCC 90,028, to 10 min for *C. krusei* ATCC 6258.

**Figure 7:**
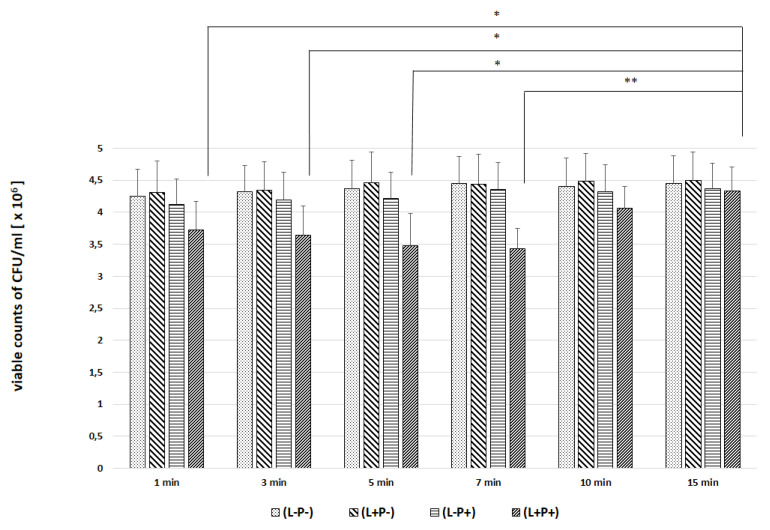
Influence of different incubation times (1–15 min) on the effect of photodynamic inactivation in reduction of number of viable cells (CFU/mL) of *C. albicans* ATCC 90,028 in planktonic form. CFU/mL was determined after TBO-mediated aPDT (L+P+), treatment with light alone (L+P-) or treatment with the photosensitizer alone (L-P+) and compared to negative control treatment (L-P-). Data are mean values and standard deviations from six replicate experiments. * *p* < 0.05, ** *p* < 0.01. *Candida* (*C.*). American Type Culture Collection (ATCC).

**Figure 8:**
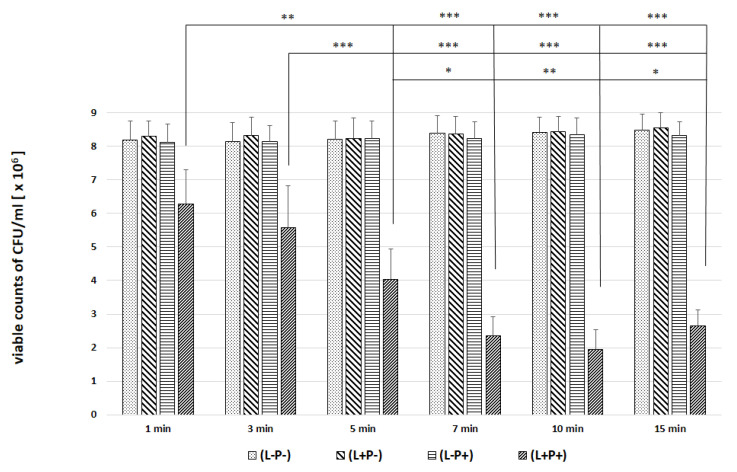
Influence of different incubation times (1–15 min) on the effect of photodynamic inactivation in reduction of number of viable cells (CFU/mL) of *C. albicans* ATCC 10,231 in planktonic form. CFU/mL was determined after TBO-mediated aPDT (L+P+), treatment with light alone (L+P-) or treatment with the photosensitizer alone (L-P+) and compared to negative control treatment (L-P-). Data are mean values and standard deviations from six replicate experiments. * *p* < 0.05, ** *p* < 0.01, *** *p* < 0.001. *Candida* (*C.*). American Type Culture Collection (ATCC).

**Figure 9:**
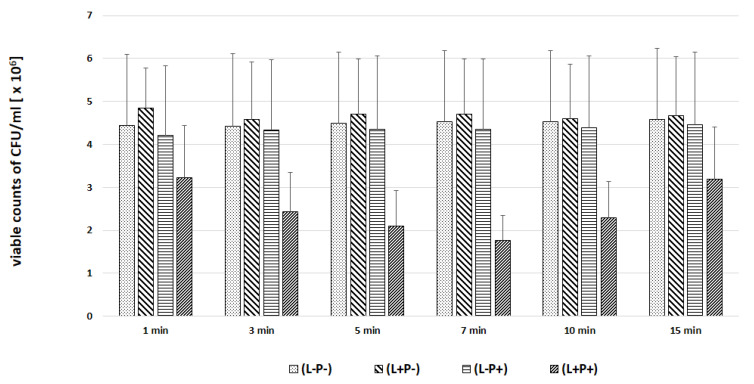
Influence of different incubation times (1–15 min) on the effect of photodynamic inactivation in reduction of number of viable cells (CFU/mL) of *C. glabrata* ATCC 90,030 in planktonic form. CFU/mL was determined after TBO-mediated aPDT (L+P+), treatment with light alone (L+P-) or treatment with the photosensitizer alone (L-P+) and compared to negative control treatment (L-P-). Data are mean values and standard deviations from six replicate experiments. *Candida* (*C.*). American Type Culture Collection (ATCC).

**Figure 10:**
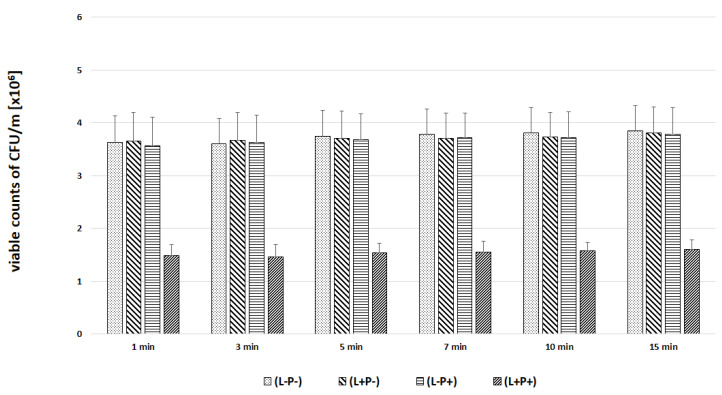
Influence of different incubation times (1–15 min) on the effect of photodynamic inactivation in reduction of number of viable cells (CFU/mL) of *C. krusei* ATCC 6258 in planktonic form. CFU/mL was determined after TBO-mediated aPDT (L+P+), treatment with light alone (L+P-) or treatment with the photosensitizer alone (L-P+) and compared to negative control treatment (L-P-). Data are mean values and standard deviations from six replicate experiments. *Candida* (*C.*). American Type Culture Collection (ATCC).

**Figure 11:**
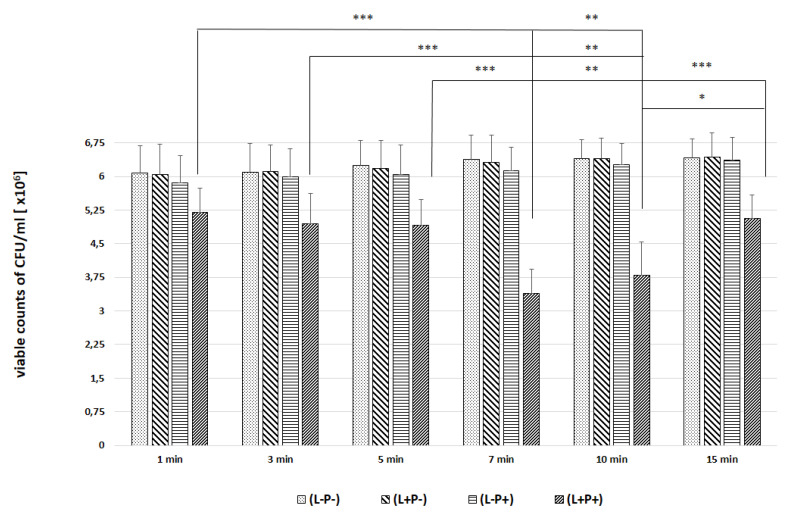
Influence of different incubation times (1–15 min) on the effect of photodynamic inactivation in reduction of number of viable cells (CFU/mL) of *C. parapsilosis* ATCC 90,018 in planktonic form. CFU/mL was determined after TBO-mediated aPDT (L+P+), treatment with light alone (L+P-) or treatment with the photosensitizer alone (L-P+) and compared to negative control treatment (L-P-). Data are mean values and standard deviations from six replicate experiments. * *p* < 0.05, ** *p* < 0.01, *** *p* < 0.001. *Candida* (*C.*). American Type Culture Collection (ATCC).

**Figure 12:**
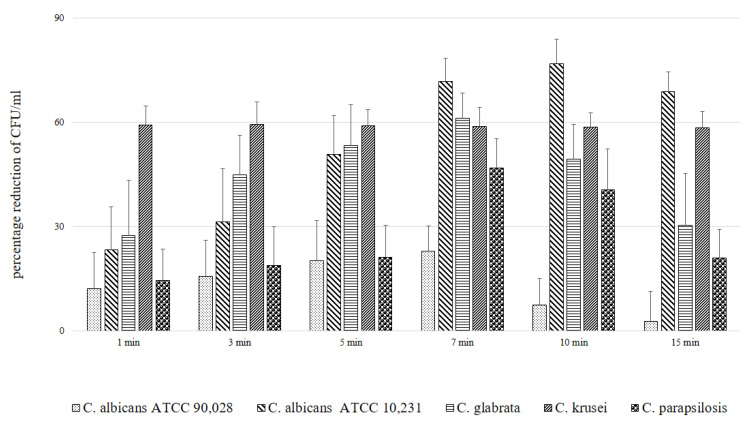
Influence of different incubation times (1–15 min) on the effect of photodynamic inactivation in % reduction of CFU/mL of *C. albicans* ATCC 90,028, *C. albicans* ATCC 10,231, *C. glabrata* ATCC 90,030, *C. krusei* ATCC 6258, *C. parapsilosis* ATCC 90,018 in planktonic form. CFU/mL was determined after TBO-mediated aPDT (L+P+). Data are mean values and standard deviations from six replicate experiments. *Candida* (*C.*). American Type Culture Collection (ATCC).

In the next experiments, an evaluation of the photodynamic inhibitory effects on the growth of planktonic *Candida* spp. cultures was made after 1, 3, 5, 7, 10 and 15 min of incubation in all test and control groups. Figure 7, Figure 8, Figure 9, Figure 10 and Figure 11 show the results as viable counts (colony-forming units per milliliter) (CFU/mL, mean ± SD). TBO-mediated aPDT significantly reduced the cell number (CFUs) of all tested *Candida* strains in comparison to the other two treatments and to the control group. The reduction depended on the time of incubation. There was no significant difference between CFU counts of groups (L-P-) and (L+P-) and (L-P+). These results indicate that laser irradiation alone or the use of the photosensitizer alone had no fungicidal effect. The highest % reduction of CFU/mL was obtained for the strain *C. albicans* ATCC 10,231 and it was 76.89%. Successively for *C. glabrata* ATCC 90,030 −61.36%, *C. krusei* ATCC 6258- 59.39%, *C. parapsilosis* ATCC 90,018 −46.81%. The lowest result was obtained for *C albicans* ATCC 90,028 and it was only 23.01% (Figure 12). Table 1 summarizes comparison of the results achieved in both parts of our experiment—the efficacy of TBO-mediated aPDT presented as the highest % reduction of CFU/mL to % of cells stained by the presence of photosensitizer in their cytoplasm visible in the microscope field for individual *Candida* strains tested.

## 3. Discussion

The initial component of photodynamic therapy protocols for efficient TBO-mediated aPDT against yeast, the optimal incubation time, appears to have a major impact on its efficacy. The results of our study confirm this impact, both by direct microscopic observation of in vivo preparations, allowing real-time evaluation of the amount of TBO absorbed by selected *Candida* strains after time intervals, as well as by the efficacy of aPDT (considered as the reduction in the number of CFU cells/mL) carried out with the same parameters of laser settings and the same PS concentration, where the only variable was the incubation time (1–15 min).

The microscopic analysis showed that for *C. albicans* ACTT 10,231, *C. glabrata* and *C. parapsilosis* the incubation time to obtain the highest number of TBO-containing stained cells was 7 min, and the remaining strains reached the maximum after 5 min. (*C. albicans* ATCC 90,018) or 10 min (*C. krusei*). However, in both cases after 7 min the mean % of stained cells was not statistically different from that achieved at the maximum (Figure 6). In the literature, only the study by Chien et al. included direct observation of *C. albicans* by confocal microscopy after 10, 30, 60 min waiting time before irradiation from the moment of mixing the suspension with 0.2 mM TBO [28]. In the present study, the amount of TBO was assessed by spectrophotometric measurement. After 10 min a large amount of dye in the cytoplasm of the cells was noted, by Chien et al. which did not significantly increase after 30 and 60 min. This observation does not correspond to our results, where in most of the strains studied (*C. albicans* 90,028, *C. glabrata*, *C. krusei* and *C. parapsilosis*) PS gradually disappeared from the cytoplasm after 5 min (Figure 1—black arrows) and 10 min (Figure 3, Figure 4 and Figure 5—black arrows) which would confirm the reports of enzymatic alteration of PS or the possible induction of drug efflux pumps by *Candida* [13,31]. We also noticed that *C. glabrata* uses an additional mechanism of protection against the effects of TBO, consisting of aggregation of individual cells into large, clustered structures that may prevent the PS from reaching cells in the center (Figure 3—black arrows). These structures appeared as early as 3–5 min after the addition of TBO to the medium (Figure 3—red arrows). A similar mechanism was not observed in the other strains.

We found only a few published studies evaluating the efficacy of aPDT with TBO in which one of the variables assessed was the incubation time [24,32,33]. In one of these, a study by Jackson et al. using a He-Ne laser with a wavelength of 632.8 nm (21 J energy) and TBO at a concentration of 25 µg/mL, it was found that the most appropriate PIT to inhibit the growth of planktonic forms of various *C. albicans* strains (including those resistant to azoles) is 5 min. A shorter time (2 min) resulted in a very poor efficacy of aPDT. Of the longer times tested (10, 60 and 180 min), the most favorable was 10 min, and the result was only slightly worse than that achieved in 5 min. In addition, it was noticed that during 180 min in the group where the effect of PS alone was assessed without laser irradiation, TBO had a detrimental effect on the viability of cells [32]. We obtained similar results in the second part of our experiments; the highest efficacy of aPDT (expressed as reduction of CFU/mL) was obtained after a 7 min incubation time (*C. albicans* ATCC 90,018, *C. glabrata*, *C. parapsilosis*) and after 10 min for *C. albicans* ATCC 10,231 and 3 min for *C. krusei* (Figure 12). For *C. krusei*, the efficacy with a 7 and 10 min incubation time did not differ statistically from that after 15 min waiting time (Figure 10). The results of the evaluation of the most favorable incubation time from both parts of our experiment coincide and indicate a 7–10 min PIT period (Table 1). The differences in the results between individual strains indicate the importance of the morphological structure of the cell wall and the resulting sensitivity of the strain to aPDT (like the phenomenon of sensitivity in pharmacotherapy).

A similar 10 min incubation time was recommended in the study by Chien et al., based on the highest efficiency of photodynamic therapy performed with the use of a 635 nm LED (fluence 50 J/cm^2^) and TBO at a concentration of 0.2 mM; extending the PIT to 30 or 60 min did not change the percentage of surviving aPDT cells [24]. Completely different results were obtained in the study by Donnelly et al. using a Paterson lamp emitting 635 nm light and TBO at a concentration of 5 mg/mL. The highest % reduction of CFU/mL for planktonic *C. albicans* was obtained with an incubation time of 30 min while a PIT of 5 min (with the same fluency of 100 J/cm^2^) resulted in a smaller but statistically significant difference [33]. The appropriate incubation time seems to be even more important in the case of experiments carried out on strains organized in a biofilm. This is connected, among other factors, with the presence of EPS (extracellular polymeric substances) which makes it difficult for a PS to reach individual cells, and with the variety of yeast strains and forms [34]. Studies show that aPDT treatment of biofilms requires longer incubation times and higher TBO concentrations, which results in improved efficacy visible as a reduction of the number of cells in both the yeast and filamentous forms [35,36] and a significant reduction in the mass of EPS (LED 635 nm, fluence 50 J/cm^2^, concentration of TBO 2.5 mM, PIT 30 min) [37]. In other studies, using planktonic solutions and biofilms of *Candida* strains with TBO-mediated aPDT, the PIT was not a variable of the protocol; the authors used 30 s to 180 min with the most common of 5 min (nine studies) [25,32,33,35,36,38,39,40,41] or 30 min (seven studies) [24,33,37,42,43,44,45]. Other incubation times used in that study were 1 min [46,47], 10 min [48] and 20 min [29]. In two other studies of TBO-mediated aPDT, the applied PIT was not reported [49,50]. All these studies showed the efficacy of the therapy as a reduction in the number of cells or % CFU/mL, but only that by Nielsen et al. showed complete elimination of *C. albicans* from the planktonic solution (LED 635 nm, 37.7 J/cm^2^, 400 mW, TBO concentration 0.226 mM, PIT 1 min) [47] and the others showed only a partial reduction. No statistically significant difference in efficacy versus control was reported only in the study by Merigo et al. carried out on a *C. albicans* biofilm (diode laser 650 nm, fluence 10 J/cm^2^, 30 mW, concentration of TBO 0.1 mM, PIT 5 min) [41]. The reductions in growth for the tested *Candida* strains in our experiment (Figure 12) are not the most effective in comparison to other published studies [36,47]. However, it was important to us in this experiment, to evaluate the incubation time itself as the only protocol variable. The results and experience will be used for other variables of the TBO-mediated aPDT protocol directed against *Candida* strains, which are the parameters of laser settings.

The literature also shows a slightly better efficacy of aPDT with TBO against *C. albicans* than against other *Candida* strains [26,32,40,42,45,46,51], especially those showing clinical resistance to azoles [24,32,40,45] despite the use of comparable experimental conditions. In both parts of our experiment also there was a noticeable difference in the percentages achieved between individual strains (Table 1): in microscopic observation (from 98.1% for *C. albicans* ACTT 10,231 to 23.6% for *C. albicans* ATCC 90,018) and after aPDT (from 78.5% for *C. albicans* ATCC 10,231 to 21.56% for *C. albicans* ATCC 90,018). This large range is probably due to the differences in structure and metabolic activity of individual strains, and a similar phenomenon is observed in the sensitivity of *Candida* strains to various antibiotics and antifungal drugs [35]. Surprisingly, the biggest difference in our results (both parts of our experiment) is that between the two different strains of *C. albicans* used. In many studies comparing the efficacy of TBO-mediated aPDT against *Candida* and bacteria, attention is drawn to the shorter incubation time (1–3 min) required for bacteria, especially Gram-positive [45,50,52,53] which is related to the different morphological structure of bacteria, the lack of membrane enzymes, and the easy access of PS [13]. In our experiments in which a commercial TBO preparation distributed for use in periodontitis and periimplantitis was used to reproduce a clinical setting, it was noted that the photosensitizer is found inside individual cells of all tested strains after 30 s and that the number of such cells gradually increases over time. However, after 3 min the % of the maximum value reached is only (*C. albicans* ACTT 10,231 −53.15%, *C. albicans* ACTT 90,018 −21.2%, *C. glabrata* −31.23%, *C. krusei* −23.7% and *C. parapsilosis* −19.0%). Therefore, when planning experiments in animal and clinical models using aPDT to treat oral candidiasis, care should be taken not to use the short incubation times recommended for bacteria.

## 4. Materials and Methods

### 4.1. Organisms and Growth Conditions

This research was carried out on reference strains of *Candida* fungi from the American Type Culture Collection (ATCC, Manassas, VA, USA): *C. albicans* ATCC 90,028, *C. albicans* ATCC 10,231, *C. glabrata* ATCC 90,030, *C. krusei* ATCC 6258 and *C. parapsilosis* ATCC 90,018. These represent the most common strains causing oral candidiasis [52]. Cultures of each strain were placed separately onto Sabouraud dextrose agar plates with addition of 4% glucose (BTL, Łódź, Poland) and incubated in atmospheric air at 37 °C. After 24 h of incubation, a sample of colonies was removed from the surface of the plate and suspended in sterile physiological solution (0.9% NaCl). The number of viable cells in suspension was counted in a spectrophotometer Densimat (bioMerieux, Marcy I’Etoile, France) at a wavelength of 950 nm and using the optical density of McFarland standard number 0.5 equivalent to 106 viable cells/mL.

### 4.2. Photosensitizer and Laser

Toluidine blue ortho—a watery solution of tolonium chloride whose concentration is proprietary information viscous fluid PAD Smart Solution, (Denfotex, London, UK)—was used for the sensitization of *Candida* strains.

The light source used was a diode laser with a wavelength of 635 nm Smart M Pro (Lasotronix, Piaseczno, Poland), mode-continuous wave (CW), output power of 400 mW, spot size of approx. 0.5 cm^2^, flat glass tip 8 mm diameter, energy density (fluence) of 12 J/cm^2^, time 30 s. These parameters were based on our previous research [46].

### 4.3. Microscopic Evaluation of the Absorption of Photosensitizer Particles by Planktonic Cells of Candida Strains in Real Time

First, 50 µL aliquots of a fresh suspension of the *Candida* strains were transferred with a sterile pipette to a glass slide and then 5 µL of TBO solution was added. A coverslip was placed on top, and excess dye was removed with an absorbent paper and towel. For all the strains, 6 replicates were performed, and images were recorded on a Nikon Eclipse 80i optical microscope at 600× magnification after 30 s and 1, 3, 5, 7, 10, and 15 min. Images of the planktonic cells of each strain without the addition of PS were used as controls. To assess the absorption of TBO by counting the % of stained cells in images corresponding to the field of view of the microscope, the original images were further processed and analyzed in ImageJ [53]; the indigo and blue areas (420–500 nm) in the original image were isolated and converted to black for better contrast to detect even small amounts of TBO before counting the cells. Cells with absorbed PS were defined as those whose cytoplasm was at least 50% full of TBO, and only those whose entire circumference was visible were counted. The counting was done manually by two independent researchers and any differences in the scores were resolved through common counting. The results were used for further statistical analysis.

### 4.4. Experimental Groups and Photodynamic Inactivation of Candida *spp*. In Vitro

In total, 720 assays were prepared, 144 for each *Candida* strain tested. These were divided into the following experimental groups: (L+P+) laser irradiation with the presence of photosensitizer (TBO) (*n* = 6); (L-P+) treated only with TBO without laser irradiation (*n* = 6); (L+P-) treated only with laser irradiation without TBO (*n* = 6); (L-P-) no exposure to laser light or TBO used as a negative control group (*n* = 6). In each of the study and control groups, the influence of different incubation times on the effect of reduction of number of viable cells (CFU/mL) were assessed as follows: 200 µL of suspension of each *Candida* strain were added using a sterile pipette to 24 flat-bottom wells of sterile, black 96-well microtiter plates with lids (Nunc, Denmark), leaving 1 well empty between successive samples to avoid cross-diffusion of light. Next, 20 µL of TBO were added to groups (L+P+) and (L-P+). In groups (L+P-) and (L-P-), 20 µL of physiological solution (0.9% NaCl) was added. Then the plates were shaken for 1 min, at 350 rpm at 35 °C in a thermo-shaker PST-60HL-4 (Biosan, Riga, Latvia). In the dark, at room temperature, the cover of the plate was removed and the wells, one by one at the right times (1, 3, 5, 7, 10, and 15 min) were irradiated according to the described protocol, in groups (L+P+) and (L+P-). During the irradiation the laser tip with the flat end (gaussian profile) was mounted on a rack just above the cell suspension (1 mm from the plate surface) and the other wells were covered with a black, matte screen with a hole whose diameter matched the diameter of the laser tip to prevent the spreading of light to neighboring regions. Immediately after irradiation, serial dilutions (10^−1^ to 10^−6^) were prepared and 100 µL of each dilution were seeded in duplicate onto Sabouraud dextrose agar with addition of 4% glucose (BTL, Łodź, Poland) and incubated for 48 h at 37 °C. After incubation, the number of colony forming units (CFU) were counted with Anacolyte automatic counter (Synbiosis, Cambridge, UK), and the colony-forming units per milliliter CFU/mL were calculated. All tests were performed 6 times and the mean values were used for further statistical analysis.

## 5. Statistical Analysis

Values were expressed as means ± standard deviation (SD). Statistical differences were evaluated by analysis of variance (ANOVA) and post-hoc comparison with the Newman–Keuls test. A *p* value of ≤0.05 was considered to indicate a statistically significant difference. The statistical analysis was performed using the software Statistica v. 7.1 PL (StatSoft, Krakow, Poland).

## 6. Conclusions

The optimal incubation time needed for the uptake of TBO by the cells of selected *Candida* strains is 7–10 min. This is confirmed both by direct observation by optical microscopy and by evaluation of the efficacy of TBO-mediated aPDT on planktonic cells of these strains.

## Figures and Tables

**Table 1:** Comparison of the results achieved in both parts of our experiment—the efficacy of TBO-mediated aPDT presented as the highest % reduction of CFU/mL to % of cells stained by the presence of photosensitizer in their cytoplasm visible in the microscope field for individual *Candida* strains tested.

Candida Strain (The Most Efficient Incubation Time)	The Highest Reduction of CFU/mL [%]	Assessment of TBO Uptake by Cells over Time [%]
C. albicans ATCC 90,028 (7 min)	23.02	19.93
C. albicans ATCC 10,231 (10 min)	76.89	94.43
C. glabrata (7 min)	61.37	40.15
C. krusei (3 min)	59.40	23.39
C. parapsilosis (7 min)	46.81	47.00

## Data Availability

Not applicable.
